# Comparative analysis of organophosphorus versus carbamate pesticide poisoning: a case study

**DOI:** 10.2478/aiht-2024-75-3781

**Published:** 2024-03-29

**Authors:** Jia-ding Xia, Hui Wang, Li-wei Hua, Min Xu, Xin Zheng, Kun Zhang

**Affiliations:** Affiliated Hospital of Chengde Medical University, Intensive Care Unit, Chengde, China

**Keywords:** acetylcholinesterase, carbamate, carbofuran, cholinergic crisis, dichlorvos, intensive care, organophosphates, acetilkolinesteraza, diklorvos, intenzivna skrb, karbamat, karbofuran, kolinergična kriza, organofosfati

## Abstract

Organophosphorus poisoning is a critical condition that can cause central nervous system depression, respiratory failure, and death early on. As its clinical manifestations closely resemble those of carbamate pesticide poisoning, the aim of this case study is to present a case of misdiagnosis, initially identifying carbofuran poisoning as organophosphate in a patient suspect of a heatstroke. We also present a case of intentional self-poisoning with organophosphate dichlorvos to underline the likelihood of pesticide poisoning in patients exhibiting acute cholinergic symptoms when the ingested substance is not known. In such cases, empirical treatment with atropine and oxime can be started pending timely differential diagnosis to adjust treatment as necessary.

## Abstract (hr)

Trovanje organofosfornim spojevima kritično je stanje koje rano može dovesti do depresije središnjeg živčanog sustava, zatajenja disanja i smrti. Budući da je prema kliničkoj slici blisko srodno trovanju karbamatnim pesticidima, cilj je ovoga članka prikazati slučaj pogrešne dijagnoze, koja je isprva trovanje karbofuranom identificirala kao trovanje organofosfatom u bolesnika u kojega se sumnjalo na toplinski udar. Usporedno dajemo slučaj namjernog samotrovanja organofosfatom diklorvosom da bismo naglasili koliko je velika vjerojatnost trovanja pesticidima u bolesnika s akutnim kolinergičnim simptomima kod kojih se ne zna progutana tvar. U takvim se slučajevima može započeti empirijsko liječenje atropinom i oksimom dok se ne ustanovi pravovremena diferencijalna dijagnoza i po potrebi prilagodi liječenje.

Intensified use of organophosphate pesticides in agriculture as replacement of banned, highly toxic pesticides such as paraquat has led to an increase in poisoning incidents in rural areas of developing countries, accounting for about 80 % of all pesticide poisonings (1,2,3). A common manifestation of organophosphorus pesticide poisoning is the cholinergic crisis, characterised by muscarinic symptoms, nicotinic symptoms, and central nervous system (CNS) depression. Clinical signs include excessive salivation, lacrimation, urination, diaphoresis, gastrointestinal disturbance, emesis, bronchorrhoea, bronchospasm, bradycardia, fasciculation, muscle weakness leading to paralysis and acute lung injury, and agitation accompanied by confusion or seizures ultimately progressing to coma. Severe cases of pulmonary oedema, central nervous system inhibition, and respiratory failure are the leading causes of death in the early stages of poisoning. Patients who survive may later develop the intermediate syndrome and delayed polyneuropathy (4,5,6,7).

Similar clinical symptoms arise from poisoning with carbamate pesticides (8, 9), which have different mechanisms of action, which is why pesticide poisoning can often be misdiagnosed as organophosphorus, especially when the agent is unknown.

The aim of this study was to present a case of a patient initially suspect of a heatstroke, then misdiagnosed with organophosphate poisoning, but whose later toxicology tests confirmed carbofuran poisoning, and compare it with a case of acute organophosphorus pesticide poisoning. We hope that this case study will provide clinicians with valuable insight for treating suspected poisoning of unknown aetiology.

## CASE REPORT

### Case 1

A 54-year-old male farmer was admitted to the emergency room (ER) over consciousness disorder accompanied by the weakness of the limbs while working outdoors in the heat. On arrival, the patient, who had had no underlying diseases, presented with dyspnoea and profuse foaming from the nasal cavity. Blood gas analysis showed hypoxaemia and hypercapnia, which immediately prompted tracheal intubation and mechanical ventilation.

Physical examination upon admission to ER showed hypothermia, normal blood pressure, stable pulse, regular breathing, lethargy, bilateral pupil diameter of 1.0 mm with moderate light reflexes, significant pulmonary wetness in both lungs, uniform heart rhythm, active bowel sounds in the abdomen, and involuntary limb movement. Laboratory tests were as follows: increased white blood cell (WBC) count (17.42×10^9^/L), prolonged activated partial thromboplastin time (aPTT) (20.00 s), low cholinesterase (CHE) (1095.47 U/L), hyperglycaemia (14.44 mmol/L), hypokalaemia (3.04 mmol/L), normal kidney function, normal B-type natriuretic peptide (BNP; 20.7 pg/mL), normal procalcitonin (PCT), normal oxygen inhalation (10 L/min), and normal blood gases (pH 7.23, PaO_2_ 56 mmHg, PaCO_2_ 64 mmHg, HCO_3_^−^ 27.4 mmol/L, lactic acid 1.5 mmol/L). Cardiac ultrasound also showed no abnormalities. Head computed tomography (CT) showed cerebral infarction. The initial diagnosis indicated that further investigation was required to determine the underlying cause of the altered state of consciousness, as the patient was suspect of heatstroke or a cerebrovascular accident.

Nine hours later the patient was transferred to the intensive care unit (ICU), where he developed coma, diaphoresis, foaming at the mouth, bradycardia (35 bpm), miosis, and fibrillation. He immediately received atropine (1 mg) intravenously, and the heart rate increased to 140 bpm, but it had to be maintained with 1 µg/min propofol infusion. Electrocardiogram (ECG) showed sinus rhythm, while physical examination revealed active bowel sounds and diminished strength and tone of the extremities.

Samples of blood, urine, and stomach contents were taken to check for poisoning. Blood CHE was 721.72 U/L. After 15 min, the patient regained consciousness and muscle strength in the upper limbs, and his pupils widened. On re-examination, blood CHE level was still low, 784.44 U/L, suggesting anticholinesterase poisoning. This prompted immediate gastric lavage, enema, and laxative administration, 10 mg atropine infusion, and intramuscular injection of 1 g of pralidoxime chloride every hour. Approximately three hours later, poison testing identified furan (carbamate) poisoning, whose blood concentration was 1.8 µg/mL, which is why pralidoxime chloride was discontinued and replaced with intramuscular injections of 0.5 mg atropine every four hours.

After about 24 h of treatment, the patient remained conscious and his muscle strength and atropine levels restored to normal. Serum CHE gradually increased from 784.44 U/L to 2424.86 U/L. Assisted ventilation was discontinued and atropine dosing lowered and eventually discontinued after 26 h of infusion. The patient’s nervous, circulatory, and liver and kidney function were normal and he was discharged from the ICU on the third day. After a one-year follow-up, the patient remained in excellent health.

### Case 2

A 68-year-old male who had been in perfect health in the past ingested intentionally 200 mL of dichlorvos and was discovered by his family about two hours later. He was immediately admitted to the Longhua County Hospital and, after failed treatment, transferred to the ER of the Affiliated Hospital of Chengde Medical College. On admission to the ER, he had been exposed to the pesticide for nine hours. He presented with consciousness disorder, sweating, salivation, incontinence, dyspnoea, nausea, and vomiting, and his breath had a characteristic odour of garlic. His body temperature was normal, pulse 130 bpm, and blood pressure low, measuring 96/54 mmHg. Propofol was administered for sedation while in the ER. The pupils measured about 4 mm bilaterally and did not respond to light stimulation. Coarse breathing was auscultated bilaterally without any signs of dry or wet rales. Heart rhythm appeared irregular, while the abdominal examination yielded negative findings. Laboratory tests showed high WBC count (14.92*10^9^/L), prolonged prothrombin time (PT, 13.90s) and aPTT (56.40 s), elevated D-dimer (6.47 µg/mL), hyperchloraemia (120.29 mmol/L), hypocalcaemia (1.73 mmol/L), high lactate dehydrogenase (343.84 U/L), procalcitonin (3.640 ng/mL), BNP (366.00 pg/mL), and low CHE (200.00 U/L). Blood gas analysis at FiO_2_ of 40 % showed pH 7.18, PaCO_2_ 34.00 mmHg, PaO_2_ 78.00 mmHg, HCO_3_^−^ 12.80 mmol/L, and base excess in the extracellular fluid (BEecf) of 15.70 mmol/L. He was diagnosed with dichlorvos poisoning and treated with gastric lavage, mechanical ventilation, haemoperfusion, and 5 mg atropine infusion every 5 min.

Upon admission to the ICU of the Affiliated Hospital of Chengde Medical College, the heart rate was 150–160 bpm, pupils gradually dilated to 7.0 mm, light reflex returned, the skin became dry, and atropine poisoning developed. The patient was given 20 % mannitol to reduce intracranial pressure, 1 mg/kg/h propofol and 0.1 µg/kg/min remifentanil for analgesia and sedation, continuous renal replacement therapy (CRRT) in combination with haemoperfusion, and intramuscular injections of pralidoxime chloride every 2 h.

As the patient developed hemodynamic instability, he was also given 0.2 µg/kg/min of norepinephrine and fluids (crystalline fluid, albumin, plasma and cryoprecipitate clotting factors) to maintain circulation.

Twenty-four hours later, the blood poison test report from the Beijing Gaoxin Hospital revealed 0.08 μg/mL of dichlorvos and 3.4 μg/mL of trimethylate phosphate, which called for continued intramuscular pralidoxime chloride injection every 4 h.

Subsequently, the patient developed a lung infection, possibly associated with aspiration, even though sputum cultures were negative for pathogenic bacteria. Considering that levofloxacin could affect pesticide metabolism, piperacillin tazobactam was administered instead to treat the infection. The patient gradually stabilised, and CRRT in combination with haemoperfusion was discontinued after four days. Vasoactive drugs were discontinued after five, and the ventilator removed after six days.

The patient requested to be transferred back to the Longhua County Hospital for further treatment and died one day later due to respiratory failure.

## DISCUSSION

Pseudo-irreversible inhibitors like carbamates do not require metabolic activation upon entering the body to induce toxicity, as they bind to the catalytic serine of acetylcholinesterase (AChE) and inhibit its activity. This inhibition leads to symptoms of cholinergic crisis resembling organophosphorus poisoning. However, unlike dephosphylation of AChE, decarbamylation is relatively fast and AChE is soon spontaneously reactivated within 24–48 h, that is, decarbamylation prevents long-lasting toxicity (9,10,11). Furthermore, whereas organophosphate pesticide poisoning can be treated with oximes with limited effect, depending on organophosphate type, poison load, and oxime dose, it is important to note that oximes are not recommended for carbamate poisoning (12). The case in point is our Case 1, which shows that pralidoxime was ineffective against carbofuran, which is why it was discontinued immediately, and we observed a significant improvement in patient’s condition within 24 h.

In contrast, organophosphates are lipophilic and can combine with body fat, leading to prolonged toxicity (13) and longer hospital stays, as observed in Case 2 (18 vs 3 days with Case 1).

### Differences in clinical manifestations and treatment of organophosphorus and carbofuran poisoning

During the early cholinergic crisis, critically ill patients may experience shock leading to severe hypoperfusion (14). Toxic substances can also damage myocardial cells and cardiac conduction, evident through arrhythmia (15,16,17). Our dichlorvos-poisoned patient described in Case 2 exhibited typical shock symptoms upon admission and developed atrial fibrillation four hours later. In contrast, the haemodynamics and cardiac function of the carbamate-poisoned patient described in Case 1 were not affected, as he did not experience arrhythmia or hypovolaemia.

Another subtle difference is that metabolic acidosis resulting from prolonged shock is more likely in organophosphate than carbamate poisoning (18). Patients with organophosphate poisoning continue to experience metabolic and lactic acidosis, which pose challenges to CRRT. The issue can be effectively addressed with pralidoxime, fluid, and sodium bicarbonate treatment, as observed by Ibrahim et al (19).

Furthermore, in addition to cholinergic crisis and shock symptoms, 10–40 % of patients with organophosphorus poisoning may develop the intermediate syndrome (IMS) within 24–96 h of the initial improvement of poisoning symptoms. The IMS is characterised by muscle paralysis primarily affecting proximal, craniofacial, and respiratory muscles and may, in severe cases, end with respiratory arrest (20, 21). However, the incidence of IMS in patients with carbamate poisoning is lower than that of organophosphorus poisoning (22).

Furthermore, the breath of most patients with organophosphate poisoning has a characteristic odour of garlic or petroleum, whereas carbamate poisoning usually does not have this particular odour (23). This distinction is evident in the two cases described above.

The diagnosis of organophosphate or carbamate poisoning is typically determined through exposure history and physical examination. However, in cases where exposure history is unknown, experience suggests the use of atropine sulphate (9, 20, 24).

## CONCLUSION

In conclusion, the clinical manifestations of carbamate pesticide poisoning closely resemble those of organophosphate poisoning, particularly during the initial cholinergic crisis. Unlike organophosphates, however, carbamates are typically odourless and more difficult to detect on breath. In both cases, treatment should start with atropine and oxime reactivators, and further course changed or adjusted as the agent is identified with laboratory tests.
